# Self-control in criminology: we need a broader conceptualization and links to psychiatric diagnoses

**DOI:** 10.3389/fpsyg.2024.1435003

**Published:** 2024-07-17

**Authors:** Sten Levander, Marie Torstensson Levander

**Affiliations:** Department of Criminology, Faculty of Health and Society, Malmö University, Malmö, Sweden

**Keywords:** self-control, self-regulation, ADHD, personality, criminality, sex, CNS functional networks

## Abstract

**Background:**

Poor self-control is a strong correlate of criminal propensity. It is conceptualized and operationalized differently in criminology than in other scientific traditions.

**Aims:**

(1) To verify the dimensionality of the criminological Grasmick self-control items, other self-regulation items and morality ones. (2) To re-interpret the dimensions using a clinical perspective, a taxonomic/diagnostic model and references to possible “biological underpinnings.” (3) Validate the dimensions by associations with crime.

**Method:**

Population: all persons born 1995 in Malmö and living there at age 12. A random sample (N = 525) filled in a comprehensive self-report questionnaire on themes like personality, crime/abuse and social aspects at age 15, 16 and 18. Age 18 data were analysed: 191 men and 220 women.

**Results:**

Self-regulation items were 4-dimensional: ADHD problems (Behavior control and Executive skills) and two Aggression factors. Morality items formed a fifth dimension. Negative Affect and Social interaction factors covered the rest of the variance. The validity of these factors was backed up by correlations with similar items/factors. Self-regulation subscales predicted crimes better than the Grasmick scale; an interaction with morality improved prediction still further. Sex differences were over-all small with three exceptions: Aggression, Morality and Negative affect.

**Conclusion:**

We identified four dimensions of the 20-item Grasmick instrument: Cognitive action control (impulsiveness/sensation seeking, response inhibition), Executive skills/future orientation, Affective/aggression reactivity and Aggression control. All should be possible to link to brain functional modules. Much can be gained if we are able to formulate an integrated model of self-regulation including distinct brain functional modules, process-and trait-oriented models, relevant diagnoses and clinical experiences of individual cases.

## Introduction

Self-control is a concept which is extensively researched in many research traditions, also with respect to its association with crime and externalized rule-breaking behavior. In these traditions self-control is a more complex concept than the criminological conceptualization by Gottfredson and Hirschi (1990), viewing it as a one-dimensional static trait. The operationalization by Grasmick et al. (1993) does not reflect the complexity of the concept, evident in the 26 chapters of the *Handbook of Self-Regulation Research, Theory, and Applications* edited by Baumeister and Vohs (2004). As stated by Inzlicht et al. (2021), self regulation is primarily a psychological concept which has proliferated into other research traditions. Thereby, integration was lost and researchers talk past each other or engage in conflicts, not the least with respect to the Grasmick conceptualisation (DeLisi et al., 2010; Moffit, 2012; Walters, 2016; Nigg, 2017; Meldrum et al., 2018; Burt, 2020).

Even if definitions and operationalization varies in the Baumeister and Vohs (2004) handbook, most of the contributors try to formulate a “general understanding.” The different theories tend to respect knowledge concerning relevant brain functional networks/modules (biological under-pinning), and are open to include clinical/typological aspects. The self-control concept in criminology appears to be detached from this general understanding. Research on crime and crime prevention is thereby hampered.

Unfortunately, criminology and many other disciplines appear to neglect the diagnostic/typological approach of clinical sciences. Few would question the statement that psychopaths display poor self-regulation and that that is backed up by thousands of research publications. The word psychopathy is not used in the in the Inzlicht, Werner et al. publication. A similar sceptizism concerns ADHD. This is unfortunate because such diagnoses provide knowledge bridges to the biological underpinnings. Why was Phineas Gage no longer Phineas Gage (www.britannica > biography > phineas-Gate)?

With respect to the brain, the most basic models of impulsivity are the clinical Utilization syndrome (L’Hermitte, 1983) and the response inhibition paradigm (Polner et al., 2015; Kolodny et al., 2020), both implicating dysfunctions in the orbito-medial parts of the pre-frontal areas (Wåhlstedt et al., 2009; Tompson et al., 2020). With respect to affect control, two systems are involved: a sub-cortical one (the primitive mammal brain, i.e., the limbic system and certain nuclei) and a cortical one with pre-frontal areas being most important (Bantjes et al., 2020). Affect incontinence is a well-known clinical phenomenon in frontal lobe dysfunction, first described scientifically by Rylander (1939). The fundamentally different functional organization in the brain of impulsivity and affect control strongly suggest that we should construct a multi-dimensional replacement of the Grasmick scale, compatible with a “general understanding” of the concept across various scientific traditions (*cf*. Bridgett, 2020; Eronen and Bringmann, 2021).

According to a process-oriented cognitive model, an impulsive system (System I) fights with a control one (System II) in behavior control in order to realize an envisiged specific outcome and avoid other outcomes (Hofmann et al., 2009, 2012). One important component in this model is executive, to formulate future consequences. The biological underpinning of the two systems is well known (Lopez et al., 2017; Koban et al., 2021).

Personality trait models are alternatives to the process-oriented ones with respect to self-regulation. CG Jung is the pioneer (Jung, 1971). Personality traits are now crystallized into five uncorrelated dimensions, the Big Five and accepted in the DSM-5 (PID-5, 2013) typological conceptualization of personality disorders. The Grasmick operationalization is claimed to be one-dimensional (idem). In the PID-5 (idem) the corresponding items/characteristics are at least 2-dimensional (Emotional lability and Disinhibition).

Walters (2016) did a meta-analysis of the concept self-control based on thirteen trait data sets. He presented one answer: *Self-control is a multidimensional construct*, and one question: *What constructs are involved?* One recent meta-analysis of the full Grasmick scale yielded six factors (Pechorro et al., 2023), which were interpreted differentially than the “general understanding.” Using advanced statistics only half of the subscales were psychometrically adequate.

The psychiatric approach is typological (diagnosis-based) and includes professionally proven (clinical) experience. The ADHD diagnosis covers signs and symptoms of poor self-regulation and are strongly associated with crime (Mohr-Jensen and Steinhausen, 2016; Anker et al., 2021). In statistical analyses binary assumtions and Baysean use of prior (including clinical) knowledge is accepted. In contrast, the psychological approach is dimensional and analyses are number-driven and often statistically advanced. There is always a large element of interpretation in such analyses, particularly factor analyses. That is another problem in a complex world: reality may not always be possible to describe by statistically clean models. That creates un-necessary barriers between scientific traditions studying the same reality.

Citing Inzlicht et al. (2021), *we need to integrate different models of selfregulation from within social and personality psychology and cognitive neuroscience* (we would like to add diagnostic knowledge). *Perhaps the clearest example of such confusion is with the term self-regulation itself.*

Criminality is empirically associated with various aspects of poor self-regulation as well as with weak moral control and their interaction (Wikström et al., 2024). *Astonglishly little is known about the individual and situational characteristics that affect the functioning of self-control in relation to crime* (Hirtenlehner and Leitgöb, 2021), i.e., the mechanism-based interplay between self-control and morality. Hence, this is important to analyse. Finally, we need to apply a life-course perspective – how different functional modules mature and link together over time. This is not a linear process: with puberty self-regulation begins to fail until age 15, then it increases (Atherton et al., 2020). Clinically there are large sex differences, partly explained by the slower myelinisation of the male brain.

We re-analysed previously collected self-report questionnaire data inspired by the Grasmick approach. Specifically, we wanted to address the issues brought up by DeLisi et al. (2010) and Walters (2016) – the dimensionality of the criminological self-control concept and the nature of its components. We used data from a Swedish longitudinal study, Malmö Individual and Neighbourhood Data Study (MINDS), which followed 525 young people during the adolescent period (Ivert et al., 2018; Chrysoulakis, 2022).

### Aims

The overall aim was to use a clinical and cross-scientific approach for re-analysing a relevant data set with a particular focus on self-regulation and its association with norm-breaking behavior and crime, specifically by adding a typological diagnostic approach and consider possible biological underpinnings (brain functional modules) of behavior.

1. Reconsider the factor structure of the Grasmick scale, establish its construct validity by documenting associations with relevant items/scales and suggest alternative interpretations of what we measure by such scales/components.
2. Construct a replacement of the Grasmick scale using an alternative factor structure, compatible with the general understanding of self-regulation and typological constructs.
3. Verify the new scale’s predictive power for norm-breaking behavior and crime and the additional predictive power via an interaction with morality.

### Method

Data were drawn from the Malmö Individual and Neighbourhood Development Study (MINDS), which is a longitudinal study of a randomly selected sample of adolescents born in 1995 and living in Malmö, Sweden, on September 1, 2007. The total sample consists of 525 adolescents (approximately 20% of the cohort). The data employed in the current study comes from the fourth wave of data collection, when the participants were at age 18. From the original sample 114 participants (74 boys and 40 girls) did not participate in the fourth wave of data collection. A drop-out analysis was conducted separate for sex, crime, and extraversion variables. This resulted in only a few significant differences. The data set comprised almost 600 self-report questions covering around 50 themes. Data were analysed for 411 participants (191 men and 220 women).

### Independent variables

We focused initially on an 8-item Grasmick scale [items described and inclusion motivated in Wikström et al. (2012)]. Items refer conceptually to impulsivity, sensation seeking, aggression control and future-orientation (executive), but is claimed to be one-factorial (Walters, 2016). Six Self-control items and six Morality items were added to the scale (Wikström et al., 2012; Table 1). In the following these 20 items are denoted as Grasmick/Self-control/Morality (GSC/M) ones. Exploratory oblique factor analyses and homogeneity analyses resulted in four subscales (Table 2).

**Table 1:** The 20 Grasmick self-control/morality (GSC/M) items.

Question	Origin	Factor	Extr.
1 When I am really angry other people better stay away from me.	G	Aggr.	0.84
2 I often act on the spur of the moment without stopping to think.	G	ADHD	0.55
3 I always react with poor conscience when I do something wrong.	M	Moral	0.67
4 I tend to become irritated or crossed on other people.	S	Orph.	0.50
5 I sometimes find it exciting to do things that may be dangerous.	G	ADHD	0.78
6 I do not devote much thought and effort preparing for the future.	G	Exec.	0.73
7 Sometimes I will take a risk just for the fun of it.	G	ADHD	0.78
8 If I do something that upsets people, it is their problem, not mine.	S	ADHD	0.57
9 I often try to avoid things that I know will be difficult.	G	Exec.	0.40
10 I do not care much if other people think that I act wrongly.	M	Moral	0.54
11 I never think about what will happen to me in the future.	G	Exec	0.71
12 I always try to avoid to hurt and harm people.	M	Moral	0.60
13 I get bored easily.	S	ADHD	0.43
14 I often feel stupid when I do something that is wrong.	M	Moral	0.70
15 I lose my temper pretty easily.	G	Aggr.	0.74
16 I always get a bad conscience when I am late paying back money to friend.	M	Moral	0.43
17 When I get angry I have difficulties to control what I do.	S	Aggr.	0.87
18 If I feel tempted to do something which I should not do, I will often do it.	S	ADHD	0.73
19 If I feel tempted to do something which I should not do, I never think about problems that might happen – be detected or be caught.	S	Orph.	0.50
20When I get angry I never think about the consequences of what I do.	S	Aggr.	0.79

Origin: G(rasmick), M(oral), S(elfcontrol). Facor 1: Aggression control. Factor 2: ADHD problems. Factor 3: Morality. Factor 4: Executive. Orphan, not used. Extraction coefficients for the factor is provided.

**Table 2:** Mean values and SD for seven personality factors of the GSC/M and SDQ questionnaires, and statistical and clinical (Cohen’s d) significance for the sex differences.

SC/M factors	Sex	Mean	SD	Sign M/F	Size
Aggression	M	5,77	3,59	N.S.	0,17
	F	5,19	3,25		
ADHD-rel.	M	6,21	2,83	*p* < 0.05	0,20
	F	5,62	3,11		
Morality	M	2,65	2,17	*p* < 0.000	0,48
	F	1,70	1,71		
Executive	M	2,25	1,89	*p* < 0.05	0,26
	F	1,80	1,66		
					
SDQ factors					
Neg. affect	M	14,8	7,44	*p* < 0.000	0,87
	F	20,0	6,54		
ADHD-rel.	M	4,85	3,22	N.S.	0.00
	F	4,85	2,80		
Social interact.	M	12,1	2,01	N.S.	0,25
	F	12,6	1,59		

The Strength and Difficulties Questionnaire (SDQ) is a clinically oriented 25-item trait instrument compiled from different sources to assess internalized/externalized reaction patterns and social aspects of children/adolescents (Goodman et al., 1998; Hagquist, 2007). The 20 + 25 GSC/M and SDQ items generated seven subscales which are the main independent variables of the present study (Table 2).

Other relevant items in the data set were considered theme-wise and analysed using the same approach (exploratory factor analyses followed by analyses of scale homogeneity, *vide supra*).

Of specific interest for the current study were items forming subscales of Future-orientation (executive), Aggression, Morality, Shame and Guilt as specified in Wikström et al. (2012). Other sets of items (themes/subscales) reflected a range of other partly relevant individual characteristics and experiences. These subscales were used to ascertain that the participants responded in a consistent way and for construct validation purposes.

### Dependent variables

Items relevant to themes of offending/crime and externalized rule-breaking behavior (ERB) as described in Wikström et al. (2012) were considered together in exploratory factor analyses. Self-reported offending was measured with nine items: shoplifting, theft from a person, assault, robbery, residential burglary, non-residential burglary, theft from/of a car, vandalism and arson. For assessing ERB we included “minor crimes” (walking against red light, stealing a pen), truancy, bullying and conflicts with parents, teachers, and peers.

A series of explorative oblique factor analyses of the self-reported crime and the ERB variables suggested a simple almost orthogonal 2-factor solution, explaining 65% of the total variance. Drug crimes were omitted, and sex crimes and partner violence (both unusual) came out separately and are not included in the following analyses. Factor 1 included all serious crimes, Factor 2 included minor crimes and ERB. Scales of Crime and of ERB were computed and highly homogenous (r_iccc_ single item was 0.44 and 0.35). The Crime subscale was highly skewed and transformed according to the following algorithm: 0, 1, 2 to 3, 4 to 9 and 9–100 crimes. 57% reported no crimes and 2% 9–100 crimes. Furthermore, we constructed a crime versatility index ranging from 0–9 crime types as described by Hare (1991).

### Analytic strategy

SPSS 26.0 package was used to assess statistical significance of differences and associations. In addition, the size of statistically significant effects was computed, whenever it was meaningful. For differences in means, Cohen’s d was used. There are similar but non-consistent definitions of effect size for correlation coefficients in the literature. We used the R index to assess shared variance - *R* > 0.039 (*r* = 0.20) as a small association, *R* > 0.11 (*r* = 0.35) as a moderately strong one and R > 0.19 (*r* = 0.45) as a strong association.

There were fewer than 3% missing values for most of the items - these were replaced by imputation, as the most common value unless a regression analysis suggested that a separately computed value should be imputed.

### Ethics statement

The project was approved by the Regional Ethics committee at Lund university 2007 and 2014 (dnr 2007/201 and dnr 2014/826).

## Results

The factor analyses of the 20 *GSC/M* items resulted in four factors keeping 50% of the initial variance (Table 2). From a clinical criminological perspective, the factors were interpreted as *ADHD problems* (ADHD-1)*, Aggression* (Aggression-1)*, Guilt/Shame/Morality* (Morality-1) *and Executive functions*. The two first factors shared 25 per cent of the variance; the others were essentially uncorrelated. Three items with no clear association to one of the factors were excluded. Four GSC/M subscales were defined according to the outcome of the factor analyses and checked for homogeneity (r_iccc_, single value >0.30). Items had similar variance; hence subscales were computed by the mean of item responses and named as in Table 2.

Exploratory factor analyses of the 25 *SDQ* items resulted in three factors, explaining 55 per cent of the total variation and interpreted as *Negative affect* (PID-5 terminology), as *ADHD problems* (ADHD-2), and as *Extraversion/Social Desirability*. Three items displayed no distinct factor association and were excluded. Subscales were computed, homogeneity was >0.30 except for the Extraversion scale for which r_iccc_ was 0.19. Scale names are given in Table 2.

A comparison of men and women with respect to the seven factors (four GSC/M and three SDQ ones) yielded the results presented in Table 2 (t-tests were used for statistical significance). Sex differences were obtained for Moral control and Negative affect.

We contrasted the GSC/M Morality (Moral-1) subscale with the 18-item *Shame & Guilt* and the 16-item *Importance of morality rules* scales [these scales and the following ones are described in Wikström et al. (2012)]. As expected, associations were strong among these three subscales. A Moral-2 subscale was based on the Shame & Guilt items, a Moral-3 subscale on the Importance items. The GSC/M Aggression subscale was contrasted with a separate 16-item Aggression-2 scale – but these scales had only 7 % shared variance. Other statistically significant associations between the GSC/M and SDQ subscales on one hand and scales based on relevant themes were obtained for most of the analyzes (details not provided). These analyzes suggest that participants responded in a consistent way to similar items, and that the subscales represent clinically/conceptually meaningful and homogenous constructs.

The Aggression-1 subscale items are formulated to reflect control, not intensity: *How well are you able to control your anger?* In contrast, the items of the Aggression-2 subscale reflects intensity and type of trigger: ‘*How angry do you become if someone …*’. The shared variance was only R = 0.07 - hence it may be meaningful to construct an index reflecting whether affective reaction intensity or failure to control the affect is the larger problem. The two aggression factors were z-transformed, and a difference score was computed. High positive values suggest that affect intensity is the larger problem, negative values suggest control problems. Boys had a mean of −0.37; girls 0.33. The difference is statistically highly significant (*t* = 5.93, *p* < 0.000) and with respect to effect size almost large (d = 0.70). Hence, girls report more problems with reactions to aggression triggers, boys with aggression control.

The Crime index was extremely skewed, this was reduced by defining subclasses, see above. The Crime and the Versatility indices were strongly intercorrelated (0.91). The ERB index was normally distributed. The Crime and the ERB indices appear to be two different things with a moderate/small intercorrelation (*r* = 0.35 for boys; *r* = 0.26 for girls). A small subgroup of individuals, somewhat more boys, committed most of the serious crimes. ERB appears to be a normally distributed youth phenomenon with statistically significant but clinically small sex differences.

Intercorrelations between independent variables and the Crime and ERB indices are shown in Table 3, separately for sex. Most correlations were significant. The overall pattern of associations is quite similar for the two sexes. However, there are some note-worthy differences, particularly with respect to ADHD problems, Aggression and Morality.

**Table 3:** Correlations between self-control/morality subscales and two crime indices, separately for sex.

Scale	Sex	Instrument	Versat.	Crime
GS	M	Grasmick 8 items	0.30	0.31
	F		0.36	0.37
Aggr-1	M	GSC/M	0.25	0.27
	F		0.32	0.34
Aggr-2	M	Aggr 16 items	0.17	0.11
	F		0.02	0.00
ADHD-1	F	GSC/M	0.35	0.36
	F		0.40	0.41
ADHD-2	M	SDQ	0.47	0.43
	F		0.40	0.38
Exec	M	GSC/M	0.16	0.10
	F		0.16	0.18
Moral-1	M	GSC/M	0.16	0.15
	F		0.27	0.31
Moral-2	M	Shame/Guilt	0.37	0.31
	F		0.38	0.40
Moral-3	M	Respect law	0.31	0.28
	F		0.30	0.27

*r > 0.15; ^**^r > 0.20; ^***^r > 0.32.

Sex-separate multiple stepwise linear regression analyses were run with the predictors in Table 3 for the Crime, Versatility and ERB indices. Predicting the Crime index, the ADHD-1 index and its interaction with Morality were selected (*R* = 0.21) for boys. For girls the same predictors were selected, and in addition the SDQ ADHD-2 scale (*R* = 0.21). The Grasmick subscale (GS) was not selected.

For Versatility, the same indices were again selected, and in addition Aggression-1 and Aggression-2 for both sexes (*R* = 0.26 and *R* = 0.21, respectively).

For ERB the sex pattern was different. For boys, the ADHD-1 vs. Morality interaction term was selected first, then ADHD-2 and finally the Aggression-2 index (*R* = 0.22). For girls, the Grasmick subscale came out first, then Aggression-2.

Summing up, the ADHD-1 index displayed sex-independent and strong associations with the two more serious crime indices (Crime index and Versatility), with a further contribution by interactions with morality. The prediction of ERB does not differ from what predicts serious crimes among boys – but is different for girls.

## Discussion

The expected 2-dimensional factor structure (self-control and morality) of the 20 GSC/M self-report items was not replicated. Hence, self-control, interpreted in the light of clinical criminological knowledge, is conceptually different from the one-dimensional Grasmick self-control scale (DeLisi et al., 2010; Walters, 2016; Nigg, 2017; Kroneberg and Schulz, 2018; Pechorro et al., 2023). The four self-control dimensions which we identified reflect, as far as we know, different sets of CNS functional networks, may have different associations (causal explanatory power) with crime and may interact differentially with criminogenic context variables.

There were some sex differences but for most of them the effect size was small. For the Morality dimension, boys were less controlled by such considerations when acting.

The four GSC/M dimensions (Aggression-1, ADHD-1, Morality-1 and Executive-1) displayed highly significant associations with other scales assessing the same constructs as well as significant associations with other scales which was expected according to theory, i.e., construct validity. Aggression-1 was linked to for instance Family climate, Dys-social friends and Conflicts with parents, teachers and peers. Hence, the four GSC/M factors appear to represent a valid summation of important clinical and crime-relevant individual characteristics.

With respect to self-reported crimes and externalized rule-breaking behavior (ERB), only two highly homogenous factors came out, covered a surprisingly large amount of the total variance and were only weakly correlated.

One of the Big five personality factors, Negative affect using PID-5 terminology, came out essentially uncorrelated with anything except sex in the present analyses. The Extraversion/Social desirability factor was only marginally homogenous and includes a conceptual mix of items. Many were identical to social desirability items in older scales (EPQ *Lie* scale and Marlowe/Crowne and KSP *Social desirability* scales). These in turn tend to be negatively correlated with psychopathy checklist scores (Levander, 1979). Paradoxically, the most skillful liers score low on the EPQ Lie subscale. This conceptual mix of items may explain the nonsignificant correlation for Extraversion/Social desirability with crime.

A more detailed analysis of the association between the GSC/M Aggression index (mainly reflecting poor affect control) and the other aggression indices suggests that it is a core individual characteristic of relevance to criminology. We were able to look at the balance between triggers and control when it generated problems. We identified an interesting sex difference – and it was almost large in respect of clinical effect size. Girls had problems by reacting too strongly to aggression-provoking situations; boys had problems to control aggression once it had appeared. A closer look at the actual items (a clinical approach) revealed that many of the aggression-provoking situations described bullying of an innocent and defence-less victim – and to these items girls reacted more strongly than boys. Is it an issue of compassion rather than aggression? With respect to the biological underpinning we know that at least two distinct functional networks are involved, one of which is partially subcortical (Bantjes et al., 2020).

We identified several sex differences with respect to the two sets of independent and dependent variables – however fewer and smaller than might be expected. Are the mechanisms leading to criminality the same across sex or should we look for specific sex-associated criminal career mechanisms?

The actual findings are fully compatible with most of the current empirical criminological studies, for instance the Situational Action Theory (SAT) assumptions regarding self-control and morality (Wikström et al., 2024). However, such concepts need to be linked to the personality trait tradition as well as the diagnostic tradition of medicine, particularly ADHD but also PTSD which is associated with poor self-regulation and aggression control (Svingen, 2023) as well as criminality (Coker et al., 2014). It is obvious that the self-control items of the GSC/M and SDQ instruments are almost identical to the DSM-5 diagnostic criteria for an ADHD diagnosis.

ADHD is a typological concept and as such strongly associated with crime (Coker et al., 2014; Anker et al., 2021). It might as well be seen as an over-populated specific corner in a multi-dimensional space. Hence, dimensional and typological analyses are fully compatible but differentially preferred in psychiatry and psychology/criminology. Multi-factorial mechanisms generate normal distributions – local aggregation suggests that a simple 0/1-coded causal mechanism operates. That mechanism, in the present context, is most probably genetic; the heritability of ADHD is very high (75%) and special by being multi-genetic, including effects of specific profiles of genes rather than single genes (Faraone and Larsson, 2019). We need a “dimensional” name corresponding to the typological ADHD concept which in this study includes four components: cognitive action control (two components: impulsiveness/sensation seeking/response inhibition, and future orientation), affective reactivity and affective control. These should be possible to link directly to corresponding and to some extent shared brain functional networks (Salum et al., 2014; Yap et al., 2021). There is a growing body of studies reporting findings similar to ours with respect to self-regulation and crime (Schoepfer et al., 2019; Krona et al., 2021). The Morality dimension is also linked with typological diagnoses: Conduct and Antisocial disorders, and with psychopathy: “They know the words but not the music” (Cleckley, 1941) and display deviant brain functionality (Johanson et al., 2019; De Brito et al., 2021). Finally, the core concepts of agency, free will and responsibility, is currently operationalized in terms of brain processing – providing new inputs to the discussion which Aristotle started 2,376 years ago (Aristotle, 2014; Lavazza and Inglese, 2023).

## Conclusion

We need to integrate knowledge from different scientific traditions, which employ either dimensional or typological approaches. We also need clinical knowledge in order to understand how to prevent crime, one of the most important problems in our time. Clinical knowledge, the typological approach and biological underpinnings have been under-used and un-integrated in criminology since many decades – the rapid development of methods to study the working brain provides us with new possibilities. Since 1999, the Freudian school wants to participate (Abrams et al., 2023). Citing Inzlicht et al. (2021) again: *It is tempting to try to combine* var*ious models of self-regulation into a single comprehensive model*. The authors concluded that it would be premature. We think that a new Handbook would be timely.

### Limitations

This is one study performed in one city in one welfare country at this specific time. More but not many more studies are needed in order to generalize the findings. Analyses cannot be done unless N is large enough and the item pool is rich and organized according to theory – virtues of the present study. Another virtue is that we have identical data sets for the same participants at age 15 and 16, yet to analyze by a clinical criminological approach.

## Data availability statement

The datasets presented in this article are not readily available. According to the ethical approvement data cannot currently be delivered to other parties. Once the last phase of data collection is complete, the data will then be anonymised. Requests to access the datasets should be directed to Marie.Torstensson.Levander@mau.se.

## Ethics statement

The studies involving humans were approved by Regional Ethics committee at Lund university 2007 and 2014 (dnr 2007/201 and dnr 2014/826). The studies were conducted in accordance with the local legislation and institutional requirements. Written informed consent for participation in this study was provided by the participants' legal guardians/next of kin.

## Author contributions

SL: Conceptualization, Data curation, Formal analysis, Methodology, Writing – original draft, Writing – review & editing. ML: Conceptualization, Funding acquisition, Methodology, Project administration, Resources, Writing – original draft, Writing – review & editing.
